# U.S. POINTER: Design of the Multidomain Lifestyle Interventions

**DOI:** 10.1002/alz70855_098286

**Published:** 2025-12-23

**Authors:** Jeff Katula, Jennifer Ventrelle, Sarah Graef, Allison Heinrich, Sharon Wilmoth, Scott Rushing, Kristin R Krueger, Martha Clare Morris, Miia Kivipelto, Heather M Snyder, Maria C. Carrillo, Laura D Baker, U.S. POINTER Study Group

**Affiliations:** ^1^ Wake Forest School of Medicine, Winston Salem, NC, USA; ^2^ Rush University Medical Center, Chicago, IL, USA; ^3^ Rush University, Chicago, IL, USA; ^4^ Wake Forest University, Winston‐Salem, NC, USA; ^5^ Wake Forest University School of Medicine, Winston Salem, NC, USA; ^6^ Wake Forest University School of Medicine, Winston‐Salem, NC, USA; ^7^ Rush Institute for Healthy Aging, Chicago, IL, USA; ^8^ Division of Clinical Geriatrics, Center for Alzheimer Research, Department of Neurobiology, Care Sciences and Society, Karolinska Institutet, Stockholm, Sweden; ^9^ Alzheimer's Association, Chicago, IL, USA

## Abstract

**Background:**

U.S. POINTER was a multisite randomized controlled trial testing the impact of multidomain lifestyle intervention on cognitive function in cognitively unimpaired older adults at risk for cognitive decline and dementia. Inspired by the Finnish Geriatric Intervention Study to Prevent Cognitive Impairment and Disability (FINGER), the study aimed to assess the generalizability of FINGER's positive cognitive findings in a diverse and representative U.S. cohort.

**Method:**

The U.S. POINTER interventions were modeled on FINGER, targeting physical activity, nutrition, cognitive/social challenge, and cardiometabolic risk management. Additional goals included (1) delivering the intervention within the community, (2) collaborating with community partners (Alzheimer's Association, community exercise facilities) to ensure/test intervention sustainability, (3) incorporating objective adherence metrics to permit responder analyses, (4) adapting the MIND diet, and (5) introducing a cognitive training program with greater potential for participant adoption. The interventions were designed and implemented using a cognitive‐behavioral framework and theory driven self‐motivation tools. Intervention fidelity was centrally monitored through data‐driven reports and annual site visits.

**Results:**

U.S. POINTER interventions included two arms, both targeting multidomain lifestyle modification but differing in intensity and accountability. Following the baseline assessment, participants were randomized to the Structured (STR) or Self‐Guided (SG) arm. The STR intervention included 38 group sessions, a structured exercise prescription using community facilities, a nutrition program modeled on the MIND diet, BrainHQ computerized cognitive training, regular cognitive/social challenge, and frequent cardiometabolic health monitoring. The SG intervention included six facilitated group sessions, general health education across all intervention domains, and gift cards to support participants’ chosen health behaviors. Adherence was tracked using objective and self‐report metrics, with central monitoring to ensure consistent implementation across sites.

**Conclusion:**

U.S. POINTER expands upon FINGER with modifications aimed at improving intervention uptake and accountability in a U.S. cohort. The study design will permit extensive analyses of adherence as it relates to cognitive response. Findings could have significant implications for future trials and for public health programs aimed at reducing Alzheimer's disease and dementia risk in older U.S. adults.

